# Novel High-Efficiency Nanocomposite Gate Design of Quantum-Dot Cellular Automata Based on Deep Learning

**DOI:** 10.1155/2022/9596165

**Published:** 2022-06-08

**Authors:** Yourun Zhu, Senlin Ren, Xiaolong Li

**Affiliations:** College of Electronic Information, Jiangsu University of Science and Technology, Zhenjiang, Jiangsu 212100, China

## Abstract

With the development of science and technology, the feature size of CMOS devices will always shrink to the limit. Therefore, some new nanodevices will eventually become substitutes for microelectronic devices. A new electronic revolution will break out. Nanoscience and technology is the high-tech frontier technology of the century and one of the main contents of scientific development in the new era. Its development will have a profound impact on other disciplines, industries, and society. Nanoelectronics is an important part of the discipline of nanoscience and technology, which represents the development trend of microelectronics and will become the foundation of the next generation of electronic science and technology. With the development of ultra-large-scale integrated circuits, the feature size of electronic devices is getting smaller and smaller and has entered the nanoscale from the microscale. When the size of the system is small enough to be compared with the wavelength of electrons, the quantum effect becomes the dominant current-carrying main factor in child behavior. While these new phenomena and new effects bring challenges to the original semiconductor devices, they also provide opportunities for the development of new devices. Evolutionary circuit design is based on cellular neural network and quantum-dot cells, designs combinational logic circuits through the evolutionary algorithm, uses the logic gate based on cellular neural network design as the population gene of evolutionary circuit design, enriches the diversity of the population, and improves the evolutionary algorithm at the same time, the success rate of the improved genetic algorithm for evolutionary circuits has been greatly improved, and the failure rate has been reduced from 14% to 2%, obtaining a faster evolution speed and improving the performance of the evolution circuit.

## 1. Introduction

In the nineteenth century, scientists discovered silicon experimentally and then began a wide range of research and applications, including the preparation of crystalline silicon and the exploration of its applications. Silicon is a special material with both metallic and nonmetallic properties, that is, a semiconductor material in the usual sense. Its discovery and research play a crucial role in the emergence and continuous development of integrated circuits. Nanotechnology is the science and technology of using atoms or molecules with a scale of 0.1 to 100 nm to manufacture substances, and nanoelectronics is the science of studying nanodevices. At present, due to the characteristics of new physical theories and unique working methods, nanoelectronics technology has caused extensive research in academia. The main purposes of studying nanoelectronics are to explore more advanced manufacturing technology to break through the physical size and technical limits of devices under traditional processes and then to prepare new nanomaterials and nanodevices with brand-new functions and, similar to traditional circuits, to explore nanodevices applications in the construction of electronic systems, thereby greatly improving the level of information storage and processing in electronic systems. The different-plane crossing in the current QCA circuit solves the problem of signal crosstalk caused by coplanar crossing, but it is quite difficult to physically realize the different-plane crossing. At present, there is no actual experimental result to realize the different-plane crossing special physical structure. The advantages and disadvantages of the evaluation circuit in the classic circuit can be evaluated by the number of devices, power consumption, and other evaluation criteria, but the QCA circuit is still an immature research content, and its evaluation criteria have not been unified. At present, there are only complexity, delay, area, power consumption, and other factors that determine the merits of this QCA circuit. Although these evaluation criteria are still very simple, a comprehensive evaluation of the overall performance of the circuit has not been made, and large-scale mass production is still difficult. Among all nanodevice research, QCA is considered to be the most promising new generation of new nanoelectronic components [1–8].

## 2. Related Work

Quantum-dot cellular automata (QCA) were first proposed in 1993 by Lent et al. Since QCA was proposed, it has received extensive attention and research from domestic and foreign researchers. In the QCA circuit, the core idea of studying the QCA circuit is to use the special properties and unique rules of the QCA itself to realize various unit devices and large-scale circuits in the classical circuit. Different from the basic logic gates in the classical circuit, QCA mainly realizes the circuit by the multiple gate, the NOT gate, and the derivative gate of the multiple gate. Since the two most typical circuit structures of the NOT gate are fixed, while the majority gate and the crossover are not fixed, the two structures are converted into corresponding structures according to the needs of the circuit structure. At present, the research on three-input and five-input majority gates is more extensive, and most of the majority gates used in circuits are these two structures. For example, Dharmendra Kumar et al. published in Microelectronics Journal 2016 a fault-tolerant majority gate that not only occupies a small area but also has incredible reliability; Sen et al. in the Journal of 2016 Computational Electronics has published a majority gate implemented by rotating cells, and the fault tolerance rate is close to 100% even in the case of a missing cell. In addition, when reducing circuit complexity, a five-input majority gate is a better choice. For example, Shadi Sheikhfaal et al. published a five-input multiple gate with low energy consumption and low complexity in Microelectronics Journal in 2015, and the use of the five-input multiple gate to realize the XOR gate can simplify the overall. Therefore, when a large-scale circuit is realized based on the exclusive OR gate, the complexity of the circuit is reduced. Sankit Kassa et al. proposed a five-input majority gate with the lowest complexity so far in the Journal of Computational Electronics in 2016 and published the verification of the physical calculation process. The above is a demonstration of the research results of the majority gate of the basic device, and then the majority of scientific researchers have launched the research and design of combinational logic circuits; for example, adders, comparators, subtractors, dividers, and other circuits have been designed and implemented. In 2006–2008, Huang et al. successively published the design concept based on orthogonal modules in the Journal of Electronic Testing-theory and Applications and Proceedings of the 18th ACM Great Lakes Symposium on VLSI and realized 2–4 lines decoder, and the fault tolerance performance is also theoretically analyzed on this decoder. High-bit current-carrying adders and multipliers were published by Cho et al. in 2009, and the advantages compared with the previous adders and multipliers were analyzed in detail. Until today, many QCA circuits designed using quantum cellular automata have been theoretically confirmed and experimentally tested. QCA circuit design has also become a key research branch of nanoelectronics, and many classic circuits such as adders, multipliers, and flip-flops have been transplanted into QCA circuits. Throughout the development of nanoelectronics technology, its technological development is based on novel physical theories and guided by the most advanced process technology. The development of electronic technology needs to break through the bottleneck of traditional physical size and technical limits, develop the structural potential of substances, and develop nanodevices with brand-new concepts. According to the current development of nanoelectronic technology, there are two main research approaches for nanoelectronic devices: one is new nanoelectronic devices such as resonant tunnel diodes, single-electron transistors, carbon nanotubes, and quantum cellular automata. The second is the assembly of molecules to form devices with certain functions, such as organic thin films and transparent oxide thin-film transistors. Although there has been a lot of progress in the design and research of QCA circuits in recent years, there are still many problems, such as the degradation of the stability of the QCA circuit caused by the coplanar cross-linking of the lines and the division of clock regions in the QCA circuit. Although there are still many problems, quantum cellular automata are still considered to be one of the next-generation electronic components with the most potential to replace CMOS technology [9–15].

## 3. Related Theoretical Methods

### 3.1. Cellular Neural Network

**Figure 1:**
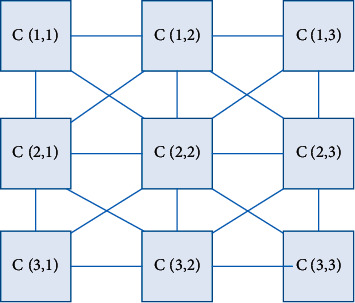
A 3 × 3 CNN structure diagram.

The structure of the cellular neural network is similar to cellular automata, each neuron is only connected to its surrounding neurons, and information can be directly transmitted between adjacent cells. They are not directly connected but can influence each other indirectly. The figure shows a two-dimensional cellular neural network. In theory, we can define cellular neural networks of any dimension but usually focus on the two-dimensional case and its application in image processing, as shown in Figure 1 [16].

For example, an *M* × *N* cellular neural network has *M* × *N* neurons, arranged in *M* rows and *N* columns; we can express the neurons in the *i*-th row and the *j*-th column as cell(*i*, *j*), abbreviated as *C*(*i*, *j*); the cell neuron in its neighborhood is defined as

(1) $$\begin{array}{c} N_{r}\text{(}i,j\text{)}=\left\{C\left(k,l\right)|\max\left\{\left|k-i\right|,\left|i-j\right|\right\}\leq r,1\leq k\leq M;1\leq l\leq N\right\}. \end{array}$$

A cellular neural network is a spatial array of locally coupled cells, each of which is a dynamical system. It consists of input variables, output variables, and state variables that change according to some prescribed dynamic laws. To be precise, it consists of two mathematical elements. (1) It consists of a spatially discrete collection of continuous nonlinear dynamical systems called cells, where each cell is loaded with information through input variables, initial states, and thresholds. (2) Each cell is centered on its own and is connected with all other cells within the radius of the sphere of influence through a coupling law.

### 3.2. QCA Cells

The basic device unit of the QCA circuit is the cell, and the cell is the basic device of the circuit, so the basic research on the circuit is the research on the cell. In the research field of QCA, first of all, from the perspective of the composition mechanism of the cell, among the currently known cells, a single cell is usually composed of two or four or five or six or even eight quantum dots and free electrons, which is the basic composition of the cell [17]. Furthermore, from the perspective of circuit operation mechanism, free electrons tunnel between quantum dots to realize information transmission, such as a five-quantum-dot cell, removing the central quantum dot, and the remaining four quantum dots are composed of four-quantum-dot cells., the operation mechanism of the two is the same, but the four-quantum-dot cell is more suitable as the basic cell of the QCA circuit, and the five-quantum-dot cell is suitable for calculating the kink energy between quantum dots. Although the above five cell structures look similar in shape, they have the same structural form, and the common point is that they all have bistable characteristics, which ensures that the cell can transmit signals correctly and effectively and effectively avoid it to solve the problem of signal weakening and crosstalk.

#### 3.2.1. Four-Quantum-Dot Cell

**Figure 2:**
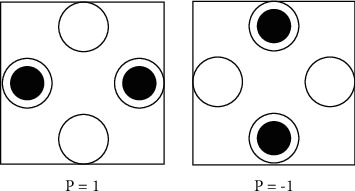
Cells in two polarization states.

A standard QCA cell of four quantum dots is mainly composed of quantum dots, electrons that can move freely in the quantum dots, and a tunnel structure. But their actual structure depends on the specific implementation. The earliest experimental verification was the cell composed of silicon-based metal semiconductors. In a standard four-quantum-dot cell, two electrons that move freely in the quantum dot can be accommodated. However, due to the high potential barrier height between cells, it is impossible for electrons to tunnel between cells, and the range of electron activity is regulated within a cell. Two free electrons tend to occupy two quantum dots on the diagonal under the Coulomb interaction. There are only two states on the diagonal in the cell. These two states are stable and unique. The two states of the cell are the characterization of the cell in the polarized state; that is, the polarizability *P* = 1 or *P* = −1, respectively, represents the “1” and logical “0” of binary information, as shown in Figure 2. This can be obtained using the polarization calculation formula, such as equation (1). In the formula, *p*1 to *p*4 represent the potential energy occupied by each quantum dot, respectively [18].

(2) $$\begin{array}{c} P=\frac{\left(P1+P3\right)-\left(P2+P4\right)}{P1+P2+P3+P4}. \end{array}$$

#### 3.2.2. Five-Quantum-Dot Cell

**Figure 3:**
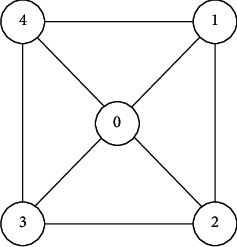
Five-quantum dot cell.

In Figure 3, the five-quantum-dot cell has one more quantum dot in the center than the four-quantum-dot cell, and we number it as 0. In order to calculate the kink energy between the quantum dots, the polarity of the cell can be calculated. Since the five-quantum-dot cell and the four-quantum-dot cell are the same, they also contain two free electrons. Since there is a quantum dot in the center of the five-quantum-dot cell, the free electrons can be transmitted diagonally, thus realizing diagonal quantum dot tunneling, which is lacking in four-quantum-dot cells. When there is no external magnetic field or electric field in the cell, the free electrons are distributed probabilistically in the five quantum dots of the cell. When there is an external magnetic field or electric field, the two free electrons show a diagonal distribution state in the cell due to the action of the external force, that is, the two cell polarization states of the above four quantum dots, which is the polarization of the cell. Its polarity calculation formula is shown in [19]

(3) $$\begin{array}{c} P=\frac{\left(P1+P3\right)-\left(P2+P4\right)}{P0+P1+P2+P3+P4}. \end{array}$$

### 3.3. QCA Basic Device Unit

#### 3.3.1. Transmission Line

**Figure 4:**
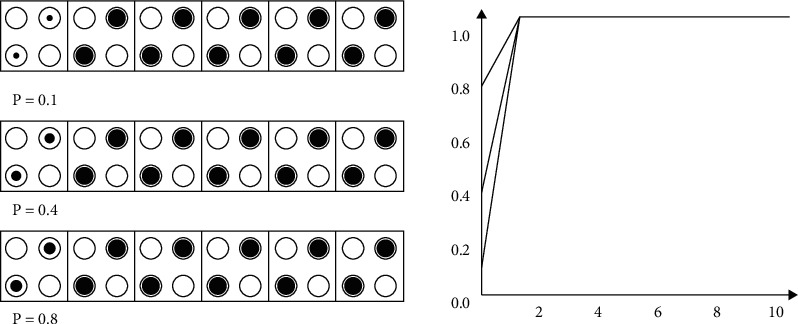
Transmission line polarization diagrams with different polarity inputs.

Transmission lines are indispensable in QCA circuits and play an extremely important role. The same or opposite information transmission is required between devices. Therefore, almost every circuit must have the participation of transmission lines. Therefore, the study of transmission lines is also particularly important, but transmission lines are also relatively simple; it has two transmission forms, namely, straight transmission line and angled transmission line, specifically as shown in Figure 4 [20].

**Figure 5:**
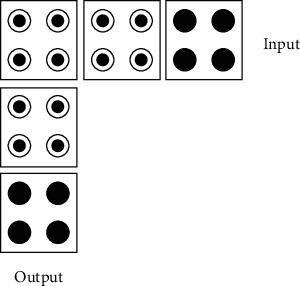
Schematic diagram of the right-angle transmission line.

When simplifying the circuit and reducing the area occupied by the circuit, the right-angle transmission line is a transmission line that must be considered. The right-angle transmission line plays a role in the information transmission of the devices in the horizontal and vertical directions.  Figure 5 is a schematic diagram of a right-angle transmission line.

**Figure 6:**
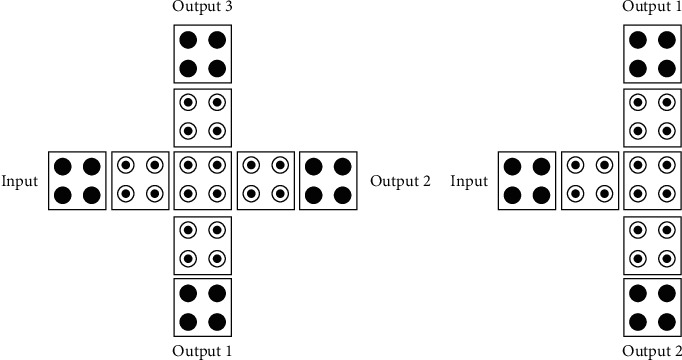
Three- and two-fan-out cables.

Angled transmission lines are often used in QCA circuits to connect two devices that are not on the same line. Sometimes, the use of angled transmission lines in circuit design will reduce the circuit area. The polarity of the cells does not change during transmission. In addition, fan-out transmission lines are used when multiplexed signals are required. Sometimes, an input signal can be used by several devices, which has to consider the fan-out transmission line, which serves as an input signal that can transmit the same signal in different directions at the same time, and the fan-out transmission line has such a function. In the QCA circuit, the fan-out transmission line has the following two structures.  Figure 6(a) shows a three-fan-out transmission line; Figure 6(b) shows a two-fan-out transmission line.

#### 3.3.2. Inverter

**Figure 7:**
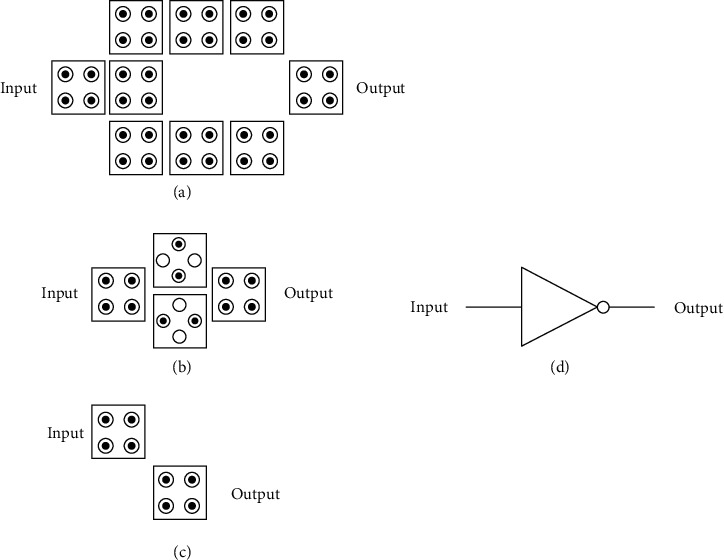
Inverter.

The inverter plays a vital role in the QCA circuit. Although there are only three structures of the inverter, the choice is very flexible when choosing its construction circuit, because it greatly simplifies the complexity of the QCA circuit degree and area, and the structure of the circuit tends to be simple. Its structure is shown in Figure 7. The following three structures are briefly analyzed.

#### 3.3.3. Three-Input Multiple Gate

**Figure 8:**
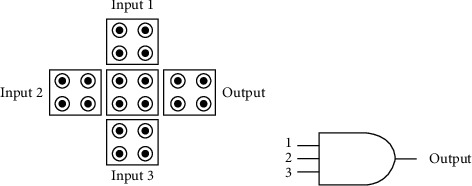
Three-input multiple-choice gate. (a) Cell diagram. (b) Logical diagram.

The QCA circuit is superior to the classical CMOS circuit. Another reason for the device is that the QCA circuit can realize the multigate structure with the least number of cells, which cannot be achieved in the CMOS circuit. Then, the multigate plays a very important role in the QCA circuit. It is a very important logic device in the QCA circuit.  Figure 8 is the cell diagram of the three-input majority gate and the logic diagram of its classical circuit.

## 4. Evolutionary Circuit Design Based on Cellular Neural Network

The circuit structure of the basic logic function of the cellular neural network is similar, and the logic function corresponding to different parameters is also different, which determines the reconfigurability of the cellular circuit. Using this property combined with the intelligent algorithm can constitute the two elements of the evolutionary circuit design. This paper mainly studies the evolutionary circuit design based on a cellular neural network and evolves the combinational logic circuit by improving the genetic algorithm.

### 4.1. Evolutionary Circuit Design Algorithm

Traditional circuit design depends on the knowledge reserve, ability, and experience of developers and the progressiveness of the design platform. When a circuit is successfully designed, its hardware circuit structure is fixed and cannot be modified. If we want to design other circuits, we need to rebuild their circuit structure, which is cumbersome and expensive. Therefore, Friedman first proposed the idea of studying evolutionary combinational logic circuits in his paper in the 1950s. He mainly proposed an idea to evolve a series of control circuit units through a “selective feedback” similar to the neural network. In the 1990s, the idea of applying this evolutionary design to circuits was called evolvable hardware (EHW).

The application of evolvable hardware enables researchers to deal with the design of more complex circuits without relying on prior knowledge and evolves design schemes that researchers cannot think of. Evolvable hardware refers to a system that uses intelligent algorithms, such as genetic algorithm (GA), evolutionary strategy (ES), particle swarm optimization algorithms (PSO), and genetic programming (GP) [30], to automatically design circuits, including reconfigurable hardware and intelligent algorithms. Evolutionary hardware has a wide range of research fields, including digital filter design, function level image filter design, robot controller design, equalizer design, combinational circuit design, sequential circuit design, embryonic electronic system design, fault-tolerant system design, analog circuit design, and polymorphic circuit design.

Evolvable hardware has two evolution situations. The first is to design circuits based on existing electronic components, such as basic components (triode, FET, resistance, inductance, and capacitance) and basic logic gates (and gate, or gate and not gate). Among them, using logic gate circuit as population design, the combinatorial logic circuit is called evolutionary circuit design. This paper studies evolutionary circuit design based on the logic gate.

The second is to find the optimal parameters of the circuit; for example, the design of a digital filter and equalizer is to find the optimal parameters fundamentally. For example, in the evolutionary design of analog circuits, when the framework of the circuit has been determined, and the important goal of its evolution is the parameters corresponding to the components, this goal is also called the design of evolutionary hardware.

At present, the main problem faced by evolutionary circuit design is scalability. Scalability is an important reason why evolvable hardware is difficult to be applied to practical engineering. It is mainly reflected in that when the input increases, the evolutionary scale increases and the time spent increases rapidly. At the same time, evolutionary algebra also increases rapidly because of the expansion of the scale of the problem. Therefore, the evolutionary ability decreases accordingly, so satisfactory results can not be obtained. At present, there are four main methods to solve the above problems: optimization of evolutionary algorithm, decomposition of input problem, improvement of “genotype-phenotype” mapping relationship, and taking function level module as the basic construction unit. The first method and the third method can partially solve this problem, and the fourth method has a certain improvement. At present, the second decomposition method is relatively good, but it requires too many resources. In general, scalability is a long-standing problem, which needs further research.

The evolution of combinational logic circuits using genetic algorithms first came from Louis' research. He combined the knowledge content of circuits with a genetic algorithm and used a masked Crossover Genetic operator to replace the classical crossover operator to optimize the evolution process. Although it can not completely solve the design problem of combinational circuits, the results also opened up a new development direction for researchers.

The genetic algorithm is a very important intelligent algorithm for computer simulation of biological behavior. It can be used for the optimization of complex systems. It has the following characteristics:

1. Genetic algorithm realizes pure mathematical operation by coding the problem. In particular, for problems that cannot be expressed by numerical values, the way of coding processing shows its advantages.
2. Genetic algorithm only converts the objective function into a fitness function and then carries out genetic and mutation operations on the fitness function without the help of other information and derivation. On the one hand, it reduces the complexity of the operation; on the other hand, it improves search efficiency.
3. Genetic algorithm has the characteristics of parallelism. The traditional optimization algorithm starts from a certain point for the iterative search. The single-point search contains less information; the search efficiency is low and even easy to fall into local optimization. The genetic algorithm starts from the whole population, which is composed of multiple individuals rather than a single individual. When the genetic operator operates on each generation of the population, it will produce a new generation of population, and there is an interactive relationship between individuals in the population. This information can be used by the genetic algorithm. Therefore, it is equivalent to searching multiple points at the same time, which speeds up the search speed.
4. The probability genetic algorithm is used. The operations of genetic operators are carried out under a certain probability, and the mutual transfer between search points is uncertain, which increases the diversity of individuals in the process of algorithm and avoids being limited to local optimization.

Using the characteristics of the circuit and genetic algorithm, the process of evolutionary circuit design is to encode the structure and parameters of the circuit, calculate its fitness function value, and observe whether the fitness value meets the requirements. If it meets the requirements, the process can be ended. Otherwise, according to the fitness value, carry out a series of operations such as cross mutation on individuals, and then repeat the above process. If the set maximum evolutionary algebra is reached in this process, the algorithm will be terminated. The coding methods include direct coding and indirect coding. This paper adopts indirect coding, which corresponds to the external evolution method. When the evolved circuit individual meets the requirements, the circuit structure corresponding to the coding is restored.

### 4.2. Improved Genetic Algorithm for Evolutionary Circuit Design

#### 4.2.1. Improvement of Selection Operator

A selection operator commonly used in basic genetic algorithms is the roulette selection operator. Due to the large selection error of the roulette wheel, degeneration sometimes occurs; that is, individuals with high fitness lose the opportunity to choose, making it difficult for the algorithm to converge to the optimal solution. Based on this, some scholars proposed a multiround roulette selection operator, let *M* be the population size, the fitness of each individual *i* is *Fi*, and the selection probability calculated according to the fitness of these *M* individuals is [0, 1] divided into *M* intervals, *ξ*_1_, *ξ*_2_,…, *ξ*_*M*_ which are *M* integers, which, respectively, represent the number of random numbers in these *M* intervals. The algorithm performs multiple rounds of roulette selection. During the multiround roulette selection process, the generated *M* random numbers are used for one round of selection, and the *ξ* values of each interval are counted to obtain *ξ*_1_, *ξ*_2_,…, *ξ*_*M*_, and take the maximum *ξ* value. The individual corresponding to the interval is the individual selected in this round, and the above operation is repeated *M* times to obtain *M* individuals.

In the above algorithm, the selection of each individual needs to perform *M* roulettes, and each generation of individuals needs to perform *M*2 roulettes. Although this method reduces the randomness of the selection, it reduces the evolution speed. Through the analysis, it can be seen that when the differences between individuals are relatively large, the use of multiple rounds of roulette selection can reduce the randomness. When the difference between individuals is relatively small, the advantage of multiround roulette selection is not significant, and since the number of roulette selections *M* is unchanged, it will greatly reduce the population evolution speed. Therefore, the number of multiround roulette selections can be appropriately reduced to increase the speed of evolution. Based on the above analysis, this paper proposes an improved multiround roulette selection operator; that is, the number of roulette rounds in each round changes with the variability of individuals. The specific number of roulettes is determined by the following formula:

(4) $$\begin{array}{c} m=\left[M\left(1-\frac{F_{\text{avg}}}{F_{\text{MAX}}}\right)\right]+1, \end{array}$$

where *m* is the number of roulettes in each generation, *M* is the population number, *F*_avg_ is the average fitness value of the current generation population, *F*_max_ is the highest fitness value of the current generation individual, and means rounding. Through this method, the number of roulette selections can be dynamically adjusted, which increases the evolution speed while reducing randomness.

#### 4.2.2. Improvement of Crossover Operator

In the basic crossover operation, individuals in the population use a fixed crossover probability for the crossover operation. However, this method of fixing the crossover probability has certain defects. As the crossover probability increases, the frequency of gene exchange between individuals also increases. However, when the crossover probability is too large, the genes of individuals with high fitness inherited will be more likely to be destroyed; if the crossover probability is too small, the frequency of gene exchange between individuals will be reduced, and the diversity of the population will be reduced, which is not conducive to jumping out local optimum. In the process of genetic evolution, the population is always changing, and the fixed crossover probability cannot adapt to the changing population. Therefore, this paper proposes a crossover operator that dynamically adjusts the crossover probability. The specific adjustment formula (5) of the crossover probability is

(5) $$\begin{array}{c} P_{c}=P_{c1}-\left(P_{c1}-P_{c2}\right)\times \left(\frac{F_{\text{avg}}-F_{\min}}{F_{\max}-F_{\min}}\right). \end{array}$$

In the formula, *P*_*c*_ represents the actual crossover probability, *P*_*c*1_ = 0.9, *P*_*c*2_ = 0.4, *F*_avg_ represents the average fitness value of all individuals in the current generation population, *F*_max_ is the highest fitness value of the current generation individual, and *F*_min_ is the current generation individual minimum fitness value degree value. From the analysis of the formula, it can be seen that when the gap between the average fitness of the population and the minimum fitness value is larger, it means that the population as a whole is concentrated near the maximum fitness value, and there are many excellent individuals. In crossover probability, when the gap between the average fitness of the population and the minimum fitness value is smaller, it means that the population as a whole is concentrated near the lowest fitness value. At this time, there are few excellent individuals, and the crossover probability should be increased to generate more excellent individuals. By adopting a crossover operator that dynamically adjusts the crossover probability, it can tend to generate more excellent individuals in order to achieve the global optimum.

#### 4.2.3. Improvement of Mutation Operator

In the basic mutation operation, individuals in the population use a fixed mutation probability for mutation operation. However, this method of fixing the probability of mutation has certain drawbacks. When the mutation probability is too large, the excellent individuals in the population are easily destroyed; when the mutation probability is too small, it is not conducive to jumping out of the local optimum. Therefore, this paper proposes a mutation operator that dynamically changes the mutation probability, which is determined by the following formula:

(6) $$\begin{array}{c} P_{m}=P_{m1}-\left(P_{m1}-P_{m2}\right)\times \left(\frac{F-F_{\min}}{F_{\max}-F_{\min}}\right). \end{array}$$

In the formula, *P*_*m*_ represents the actual mutation probability, *P*_*m*1_ = 0.1, *P*_*m*2_ = 0.001, *F* represents the fitness of the current individual, *F*_max_ is the highest fitness value of the current generation individual, and *F*_min_ is the lowest fitness value of the current generation individual. It can be seen from the formula that the larger the individual fitness is, the smaller the mutation probability is, and the easier it is for the excellent individual to be retained; the smaller the individual fitness is, the larger the mutation probability is, and the easier the population is to jump out of the local optimum. By using a mutation operator that dynamically adjusts the mutation probability, the population can be kept from falling into the local optimum while retaining excellent individuals, and finally, the global optimum can be achieved.

### 4.3. Analysis of Experimental Results

The Ackley function is used as the objective function, and the function has a minimum value, but the genetic algorithm used in this paper operates on the principle that the larger the fitness value, the better, so it is necessary to convert the minimum value of the objective function to the maximum value of the fitness function and, at the same time, consider the selection. The operator requires that the fitness values are all positive numbers, so the fitness function is selected as

(7) $$\begin{array}{c} F=20-\text{ACKley}\left(x_{1},x_{2}\right). \end{array}$$

When (*x*_1_, *x*_2_) = (0, 0), the Ackley function obtains the minimum value of 0, and the fitness function obtains the maximum value of 20 at this time. The value range of *x*_1_ and *x*_2_ is [−10, 10]. At this time, the maximum value of the Ackley function does not exceed 20. Therefore, the fitness function takes 20 and the inverse of the function to ensure that all fitness values are positive numbers. In the genetic algorithm, the population size is 50, and the terminating evolutionary generation is 100. In the basic genetic algorithm, the roulette selection method is used as the selection operator, the single-point crossover is used as the crossover operator, and the nonuniform mutation is used as the mutation operator. Select a fixed value of 0.7 for crossover probability and 0.01 for mutation probability. From this, the change curve of the fitness function can be obtained as shown below. In the 100th iteration, *x*_1_ = 0.0147, *x*_2_ = −0.1026, and the corresponding optimal fitness value is 19.4440.

**Figure 9:**
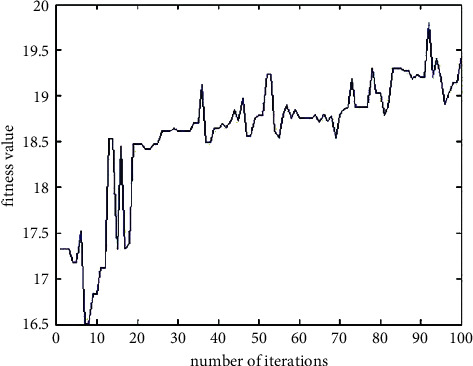
Basic genetic algorithm fitness curve.

**Figure 10:**
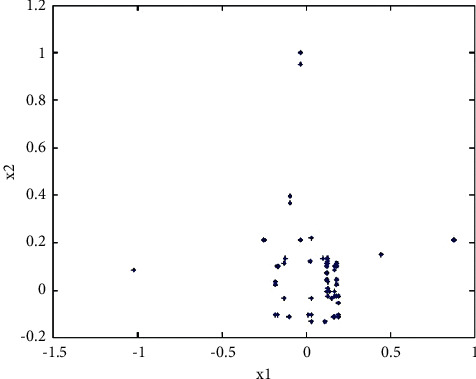
Distribution of input variables *x*_1_ and *x*_2_ of basic genetic algorithm.

It can be seen from the fitness value curve in Figure 9 that the basic genetic algorithm is not easy to retain the optimal individual, the fitness value oscillates obviously, it is easy to fall into the local optimum, and the convergence accuracy is poor. The values of the variables *x*_1_ and *x*_2_ corresponding to the fitness function are shown in the following figure. It can be seen from Figure 10 that there are many value points near the value (0, 0) range, but they have not converged to one point. The center of the circle in the figure is the location of the optimal value (0, 0). The nearby values are not completely concentrated in the circle and are scattered around the circle so that the optimal value cannot be obtained. Therefore, it proves the poor convergence of the basic genetic algorithm.

Apply the improved genetic algorithm to the Ackley function by setting the operator parameters of the genetic algorithm. Also take the population size as 50 and the number of termination iterations as 100. The fitness curve of the improved genetic algorithm can be obtained as shown below, in which, at the 100th iteration, *x*_1_ = 0.0049, *x*_2_ = −0.0049, and the corresponding optimal fitness value is 19.9792.

**Figure 11:**
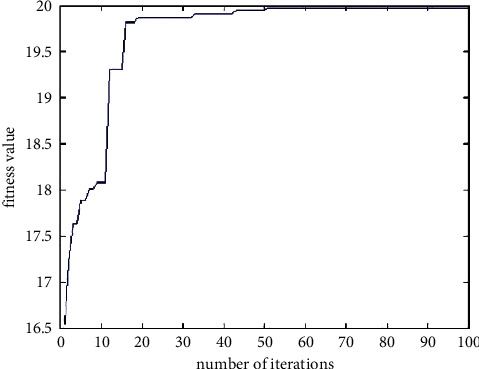
Improved genetic algorithm fitness curve.

**Figure 12:**
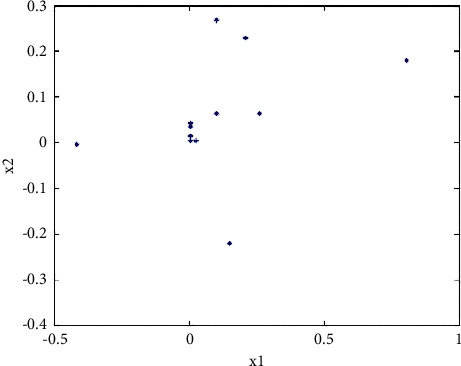
Distribution of input variables *x*_1_ and *x*_2_ of the improved genetic algorithm.

It can be seen from Figure 11 that in the 20th iteration, the optimal fitness is already around 19.8. From the overall change trend of the curve, it can be seen that the fitness is rapidly approaching the global maximum value and continues to approach the global maximum value. The maximum error is very small. It can be seen that the improved genetic algorithm has no obvious oscillation, the global convergence is good, and the speed is fast. It can jump out of the local optimum and achieve the global optimum. The values of the variables *x*_1_ and *x*_2_ corresponding to the fitness function are shown in Figure 12. Compared with the basic genetic algorithm, the number of points shown in the figure is very small. It can be seen that the convergence speed is fast, near (0, 0), and they are all in the circles in the figure. Therefore, it can be verified that the improved genetic algorithm has good convergence and high precision.

#### 4.3.1. Evolving a One-Bit Full Adder

The most basic components of a computer are basic logic gates, bit-by-bit arithmetic addition operations, and computational storage units. Among them, addition is the most basic form of computer operation, and the adder is the most basic combinational logic circuit, which can be used for binary subtraction, multiplication, and other operations, and arithmetic operations in the digital system are all performed by addition. Therefore, a full adder is analyzed and evolved to verify the superiority of the improved genetic algorithm. The experiment uses a 4 × 4 matrix to implement a one-bit full adder. The number of populations is 50, and the maximum number of generations is set to 1000. The basic genetic algorithm parameter setting is as follows: crossover probability is 0.7; mutation probability is 0.01; improved genetic algorithm parameter setting is dynamic change.

Through two genetic algorithms, run 50 times, respectively, the performance of the algorithm is shown in Table 1.

From Table 1, it can be concluded that the success rate of the improved genetic algorithm for the evolution circuit is greatly improved, and the failure rate is reduced from 14% to 2%. It is determined by experiments that the rate ratio of the basic genetic algorithm and the improved genetic algorithm is 1 : 1.24 under the same number of iterations. Compared with the basic genetic algorithm, the optimal convergence algebra of the improved genetic algorithm is reduced by 37.94%, so the overall speed is increased by 23%. The fitness value curve for one of the evolutionary processes is shown in the figure. The analysis shows that after the basic genetic algorithm reaches the maximum fitness value of 875, the improved genetic algorithm achieves the maximum fitness value of about 535. It can be seen that the convergence speed of the improved genetic algorithm is accelerated.

## 5. Conclusion

This paper is based on the evolutionary circuit design of the cellular neural network. Firstly, the principle of evolutionary circuit design and its algorithm are expounded, the performance of the genetic algorithm is analyzed, and the genetic algorithm is determined as the algorithm for evolutionary circuit design; then, the three-input linear distributable function is added as the population gene to improve the diversity of genes and enrich circuits. The diversity of evolution improves the effectiveness of the circuit; then, an improved genetic algorithm is proposed, which improves the selection operator, the crossover operator, and the mutation operator, and compared with the basic genetic algorithm, the failure rate of the improved genetic algorithm for the evolutionary circuit is reduced from 14% to 2%, the optimal convergence algebra is reduced by 37.94%, and the overall speed is increased by 23%, so as to improve the evolution rate and the overall performance of the circuit. Finally, the improved genetic algorithm is used to evolve a one-bit full adder and two-bit multiplier, and a more simplified circuit design is obtained.

## Figures and Tables

**Table 1:** List of algorithm performance parameters.

Algorithm choice	Number of runs	Average fitness	Best convergent algebra	Failure rate (%)
Basic genetic algorithm	50	13.238	854	14
Improved genetic algorithm	50	14.562	530	2

## Data Availability

The dataset can be accessed upon request.
